# QueF nitrile reductases: Catalytic mechanism and advances in biocatalyst development

**DOI:** 10.1002/pro.70838

**Published:** 2026-11-05

**Authors:** Chenlu Li, Roland Kulanda, Sam Staniland, Daniele Castagnolo, Michele Crotti

**Affiliations:** ^1^ Department of Chemistry University College London London UK; ^2^ Manufacturing Futures Lab University College London London UK; ^3^ Oncology R&D AstraZeneca Cambridge UK

**Keywords:** NAD(P)H‐dependent, nitrile reductase, nitrile reduction, primary amines biocatalytic production, QueF

## Abstract

Nitrile‐to‐amine reduction is a key transformation in organic and industrial synthesis, providing access to pharmaceutical, agrochemical, and materials manufacturing relevant primary amines. QueF nitrile reductases represent a unique class of biocatalysts capable of catalyzing the NAD(P)H‐dependent four‐electron reduction of nitriles under mild conditions. Despite their biocatalytic potential, the practical application of QueF enzymes is severely constrained by an exceptionally narrow substrate scope. This review presents a comprehensive analysis of QueF research, from its discovery, structural and mechanistic elucidation to the most recent protein‐engineering efforts. Experimental evidence indicates that the long evolutionary optimization of the active site, characterized by a highly specialized substrate‐binding pocket, is the primary determinant of QueF's substrate specificity. A critical analysis of the relationships between key active‐site residues and substrate functional groups, integrating structural, mutagenesis, and computational insights, rationalizes selectivity trends. Ultimately, this work outlines the fundamental challenges associated with expanding QueF substrate tolerance and identifies plausible strategies for future engineering. Continued progress will rely on a deeper understanding of catalytic mechanism, active‐site dynamics, and coordinated multi‐residue redesign, positioning QueF as a valuable model system for the development of next‐generation nitrile‐reducing biocatalysts.

## 1 INTRODUCTION

Amines are an exceptionally important class of compounds in organic chemistry, chemical biology, and medicinal chemistry. Their inherent nucleophilicity and reactivity make amines versatile synthetic handles and essential functional motifs in bioactive molecules such as natural products and pharmaceuticals (Bagal & Bhanage, 2015). Amines are regarded as indispensable synthons across the pharmaceutical (Afanasyev et al., 2019), agrochemical (Huang et al., 2024), and polymer industries (Chen et al., 2024). Their dual synthetic and biological relevance has triggered extensive development of catalytic methodologies for their preparation. Recent examples include palladium‐catalyzed amination of aryl halides (Hartwig, 2006; Huang & Buchwald, 2001; Lee et al., 2001), hydroaminations and hydroaminomethylation of olefins or alkynes (Ahmed et al., 2003; Müller & Beller, 1998), and homogeneous reductive aminations or direct amination of alcohols (Gunanathan & Milstein, 2008; Pingen et al., 2010; Ye et al., 2014). While this has enabled significant methodological advances, these procedures suffer limitations in substrate reactivity, functional‐group tolerance, and sustainability, and often require precious metals, harsh reaction conditions, or protective‐group manipulations. Among the different amine classes, primary amines are particularly valuable targets, both as key intermediates in synthesis and as prominent structural motifs in bioactive molecules. Consequently, the conversion of nitriles to primary amines has emerged as an attractive transformation, and hydrogenation remains the dominant industrial route for their production owing to its high atom efficiency, the relatively low cost and broad availability of organic nitriles, making the transformation attractive from a green chemistry perspective. However, these reductions typically rely on high‐pressure hydrogenation over heterogeneous catalysts (e.g., Raney Ni or noble‐metal catalysts such as Pd, Pt, or Ru or alternative systems based on Co, Sm, and In) (Bagal & Bhanage, 2015; Huber, 1944; Schwoegler & Adkins, 1939) or on stoichiometric hydride reagents such as LiAlH₄ (Amundsen & Nelson, 1951; Nystrom & Brown, 1948), which can require specialized pressure equipment, involve hazardous reagents, and generate significant waste during downstream work‐up. As prominent examples, the nitrile‐to‐amine route is a cornerstone for the production of the investigational antihypertensive nepicastat, the antidepressant venlafaxine (Saravanan et al., 2011), and the antiallergic epinastine (Scheme 1) (Park et al., 2020; Walther et al., 1990), as well as amine‐based household products such as fabric softeners and lubricants (Hinzmann & Gröger, 2020).

**SCHEME 1:**
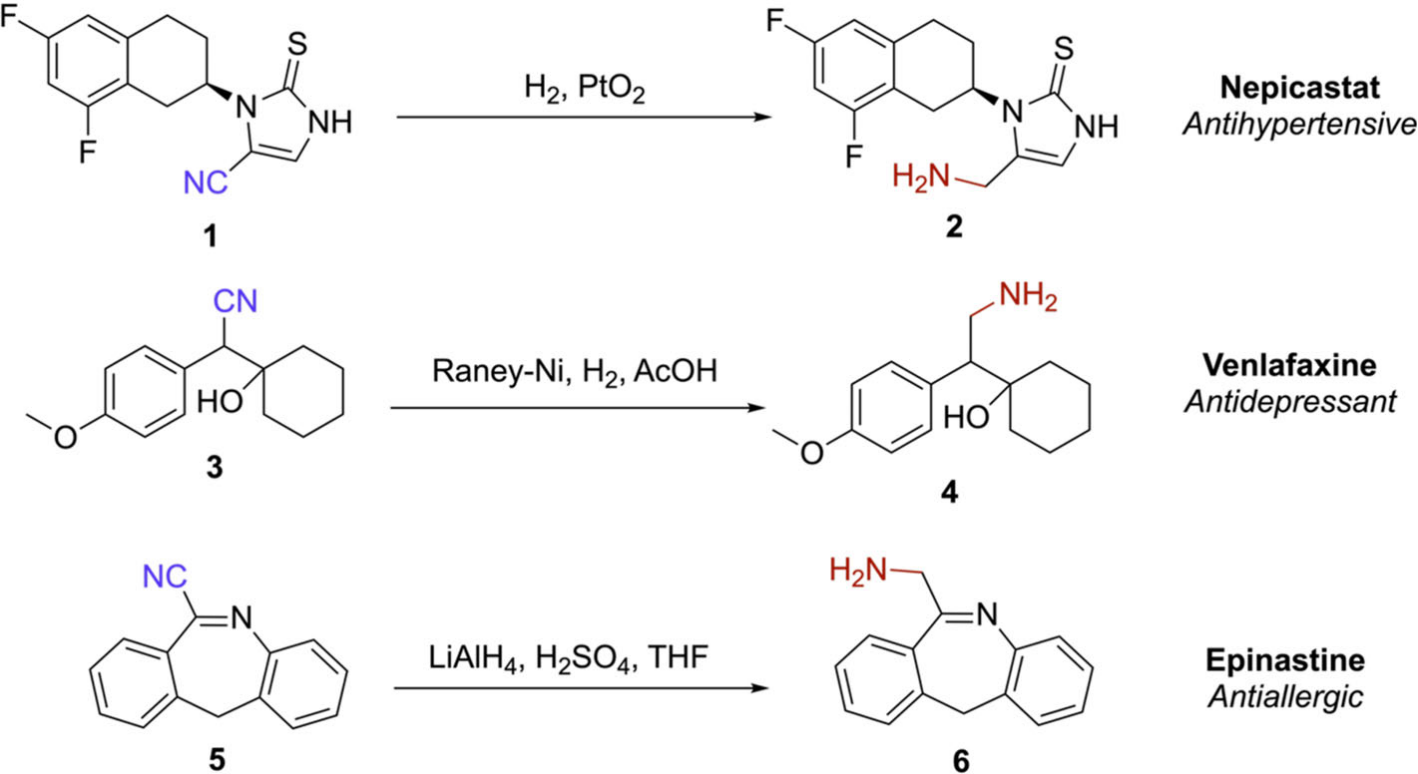
Examples of nitrile‐to‐amine catalytic industrial processes.

However, mounting pressure to decarbonise the chemical and pharmaceutical manufacturing sectors has increasingly driven efforts to replace industrially relevant transformations with more benign alternatives (Vikhrankar et al., 2024). Biocatalytic routes are widely regarded as a green and viable approach in this context, offering a complementary platform that mitigates several limitations of conventional catalysis, including reliance on precious metals, harsh reaction conditions, energy‐intensive processes, by‐products formation, and low reaction selectivity. Continued research in biocatalysis and protein engineering is broadening the biocatalytic toolkit available, and industrial adoption is growing, progressively unlocking greener alternatives for key chemical transformations (Falcioni et al., 2025). To this end, QueF nitrile reductases (NiREDs) have emerged as promising candidates for converting nitriles to primary amines under environmentally benign conditions, providing an alternative to stoichiometric and metal‐catalyzed methods (Swairjo et al., 2005; van Lanen et al., 2005). To date, QueF is the only known enzyme family capable of catalyzing a direct nitrile‐to‐amine reduction. Notably, the evolutionarily highly conserved active site confers to QueF enzymes strict substrate specificity, limiting their industrial applicability. Recent protein engineering and mechanistic studies have focused on expanding their substrate scope, and understanding the reasons underpinning this phenomenon (Wilding et al., 2012; Wilding et al., 2013). Exploration of the active sites of these enzymes has identified binding and catalytic residues, and conserved motifs that govern cofactor and substrate recognition (Chikwana et al., 2012; van Lanen et al., 2005). Despite the unique chemistry and biocatalytic potential of QueF, its development as a nitrile‐reducing biocatalyst has been scarcely reviewed, with previous work primarily examining structural and mechanistic aspects relevant to biocatalyst development (Yang et al., 2014). Since then, substrate‐selectivity and protein‐engineering studies have expanded our understanding of this enzyme family, but a systematic analysis linking the structural determinants of non‐natural substrate selectivity with engineering outcomes is still lacking. This review therefore summarizes QueF discovery and evolutionary history, current knowledge of the catalytic mechanism and substrate scope, and recent developments in protein engineering, integrating these findings to rationalize past engineering efforts and thereby provide a framework for developing variants with expanded synthetic utility.

## 2 DISCOVERY AND CHARACTERIZATION OF THE QueF FAMILY: A UNIQUE CLASS OF NITRILE‐REDUCING ENZYMES

7‐Cyano‐7‐deazaguanine reductase (QueF; EC 1.7.1.13) is an NADPH‐dependent oxidoreductase that catalyzes the unprecedented biological reduction of a nitrile functional group (van Lanen et al., 2005). Specifically, QueF converts the natural substrate 7‐cyano‐7‐deazaguanine (preQ₀) into 7‐aminomethyl‐7‐deazaguanine (preQ₁), a key step in the queuosine biosynthetic pathway (Scheme 2). This reaction defines QueF as a unique class of enzymes with no close functional analogues in primary metabolism.

**SCHEME 2:**
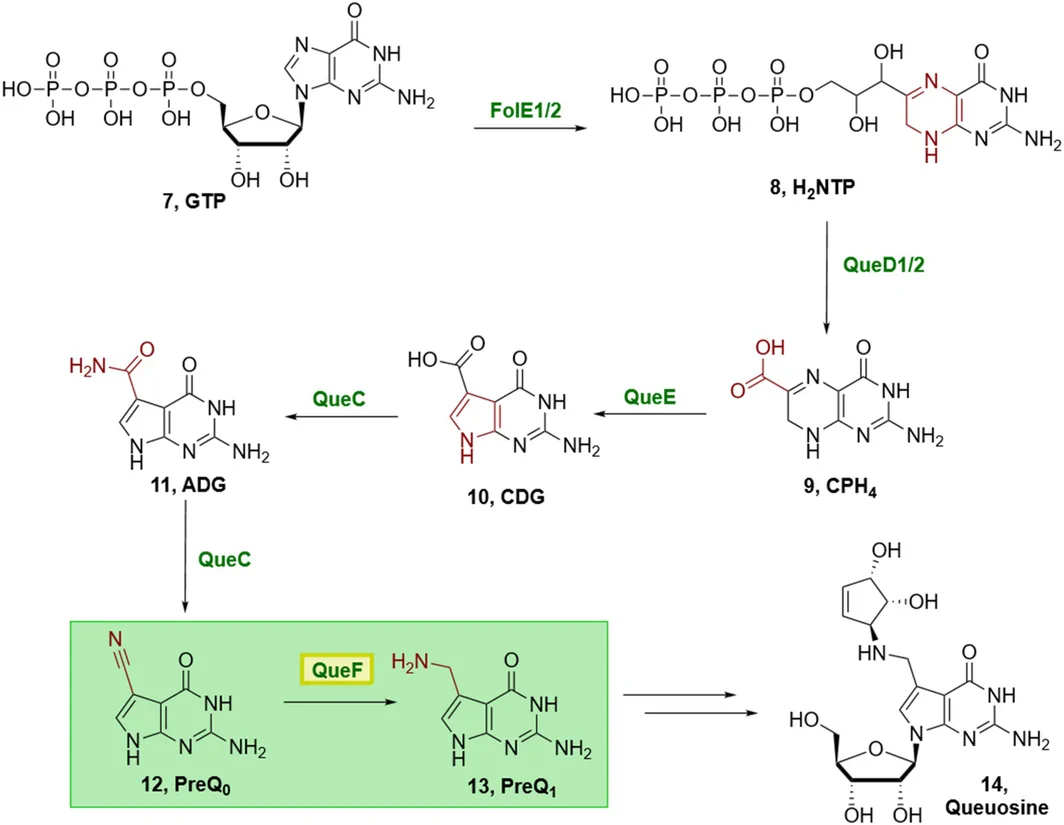
Queuosine biosynthesis from GTP, with the preQ_0_‐to‐preQ_1_ reduction‐step highlighted in green. Enzymes: FoIE1/2 (GTP cyclohydrolase IA/IB), QueD1/2 (6‐carboxy‐5,6,7,8‐tetrahydropterin‐synthase), QueE (7‐carboxy‐7‐deazaguanine synthase), QueC (7‐cyano‐7‐deazaguanine synthase) and QueF (7‐cyano‐7‐deazaguanine reductase). Intermediates: H2NTP (7,8‐dihydroneopterin triphosphate), CPH4 (6‐carboxy‐5,6,7,8‐tetrahydropterin), CDG (7‐carboxy‐7‐deazaguanine), ADG (7‐amido‐7‐deazaguanine), preQ_0_ (7‐cyano‐7‐deazaguanine), and preQ_1_ (7‐aminomethyl‐7‐deazaguanine).

Queuosine is a hypermodified nucleoside located at the wobble position (position 34) of tRNAs bearing 5′‐GUN‐3′ anticodons and is widely conserved among bacteria and eukaryotes (de Crécy‐Lagard et al., 2024). Following its insertion into tRNA and subsequent modification steps, queuosine enhances codon–anticodon interactions and modulates translational efficiency.

PreQ_0_ is a central precursor of queuosine and numerous other naturally occurring 7‐deazaguanine–containing nucleosides. It is biosynthesized from guanosine 5′‐triphosphate (GTP) through a four‐step pathway involving the coordinated action of the enzymes QueD, QueE and QueC, followed by QueF‐mediated reduction of preQ_0_ to preQ (de Crécy‐Lagard et al., 2024). The pathway is initiated by GTP cyclohydrolase I (GCYH‐I; FolE), which converts GTP to 7,8‐dihydroneopterin triphosphate (H_2_NTP). GCYH‐I belongs to the tunnel‐fold (T‐fold) superfamily, whose members share a characteristic structural architecture despite catalyzing chemically distinct reactions on related purine‐ and pterin‐derived metabolites. Schimmel, de Crécy‐Lagard, and co‐workers identified the genes required for bacterial queuosine biosynthesis providing the genetic basis for the subsequent biochemical characterization of QueF. (de Crécy‐Lagard et al., 2024; Reader et al., 2004) PreQ_1_, the reduced product of preQ_0_, represents a key intermediate in this pathway, as it can be incorporated into DNA and tRNA by tRNA‐guanine transglycosylase (TGT) enzymes (Okada et al., 1976; Okada et al., 1979). This capacity underscores the biological importance of QueF in enabling access to 7‐deazaguanosine‐modified nucleosides. (de Crécy‐Lagard et al., 2024) Interestingly, comparative genomic analyses revealed significant homology to GTP cyclohydrolase I (GCYH‐I), which led to its initial classification as a putative GCYH‐I homologue (Reader et al., 2004). Despite this homology, no cyclohydrolase‐type activity was observed for QueF (van Lanen et al., 2005), but instead its NADPH‐dependent reduction of preQ_0_ to preQ_1_ via hydride transfer from NADPH.

Nitrile‐containing metabolites associated with the queuosine biosynthesis pathway have been extensively studied in five bacterial species: *Bacillus subtilis* (van Lanen et al., 2005), *Escherichia coli* (Moeller et al., 2013), *Vibrio cholerae* (Kim et al., 2010), *Geobacillus kaustophilus* (Wilding et al., 2012), and *Pectobacterium carotovorum* (Li et al., 2016). QueF enzymes from *Bacillus subtilis* (*bs*QueF) and *E. coli* (*ec*QueF) were first cloned and characterized by Iwata‐Reuyl, de Crécy‐Lagard and co‐workers, who demonstrated that QueF catalyzes the NADPH‐dependent, four‐electron reduction of preQ_0_ to preQ_1_, identifying it as the first biological nitrile reductase (van Lanen et al., 2005). Subsequent crystallographic work by Swairjo and co‐authors reported the first crystallographic data on *bs*QueF (Swairjo et al., 2005). This was followed by mechanistic studies elucidating the enzymatic mechanism, and confirming covalent catalysis via an active‐site cysteine‐bound intermediate (Lee et al., 2007). Dynamic features such as the substrate binding‐triggered (rather than thioimidate formation‐) active‐site closure, stabilization of the functional decamer of *bs*QueF (Chikwana et al., 2012), a gating mechanism found in *ec*QueF, were characterized across the QueF family (Jung & Nidetzky, 2018). Building upon these findings, Klempier and co‐workers used computational modeling of the substrate binding‐site and site‐directed mutagenesis of *ec*QueF to investigate the enzyme's extreme substrate specificity (Wilding et al., 2013). An extensive network of pocket‐specific interactions was identified as the main source of specificity for the natural substrate preQ_0_, paving the way for future structure‐guided engineering. In a separate study, *bs*QueF was shown to form a reversible intramolecular disulfide between Cys55 and the invariant Cys99 under oxidative conditions, protecting the catalytic cysteine from over‐oxidation (Mohammad et al., 2017). Later work from Nidetzky and coworkers identified an active‐site gating mechanism found in *ec*QueF involved in the sequestration of a reactive imine intermediate (Jung & Nidetzky, 2018).

Cunningham, Joachimiak and co‐workers published the first high‐resolution crystal structure of *V. cholerae* QueF (*vc*QueF), together with simulation‐guided models of substrate and NADPH binding, supporting the catalytic mechanism elucidated in other QueF enzymes (Kim et al., 2010). Complementary QM/MM studies by Fernandes, Liang, Hirao and co‐workers on *vc*QueF subsequently proposed a detailed mechanistic description of the catalytic pathway, in agreement with available experimental observations (Ribeiro et al., 2015).

The first thermophilic QueF (*gk*QueF) was cloned and characterized from *Geobacillus kaustophilus* by Klempier and co‐authors, uncovering a pronounced temperature dependence with a 12‐fold increase in specific activity between 25 and 65 C towards preQ_0_, reaching ~0.08–0.09 U mg^−1^ at the highest temperature tested under the reported assay conditions (Wilding et al., 2012). Steady‐state kinetic analysis at 55°C revealed that *gk*QueF reduces preQ_0_ with a turnover number of 3.9 min^−1^ ± 0.6 min^−1^, corresponding to a catalytic efficiency (kcat/Km) of 0.35 min^−1^ μM^−1^. Consistent with its thermophilic origin, the enzyme also exhibited substantial thermal stability, with a half‐life of ~43 h at 55°C, which decreased to ~15 h in the presence of TCEP. Notably, a crystal structure of *gk*QueF has not yet been reported. More recently, Zhang, Xu and co‐workers identified and biochemically characterized QueF from *Pectobacterium carotovorum* (*pc*QueF) which displayed a markedly higher catalytic activity than previously reported QueF enzymes (Li et al., 2016). Under identical assay conditions, *pc*QueF exhibited a specific activity of 0.366 U mg^−1^, ~2.6‐fold higher than that of *gk*QueF (0.140 U mg^−1^), while its half‐life was 13.6 h at 40°C. Following site‐directed mutagenesis, H224N *pc*QueF variant showed a further increase in specific activity to 0.526 U mg^−1^, together with improved thermostability, as reflected by a half‐life of 32 h at 40°C (Zhou et al., 2018).

## 3 CONSERVED STRUCTURAL FEATURES AND FOLDING

Similarly to GCYH‐I GTP cyclohydrolases, QueF belongs to the tunnel‐fold (T‐fold) superfamily, which comprises enzymes catalyzing a variety of reactions on closely related purine and pterine‐type substrates (Chikwana et al., 2012; Colloc'h et al., 2000). These T‐fold domains typically span 120–160 residues and adopt a *ββααββ* topology: four antiparallel β‐strands forming a twisted *β*‐sheet with two antiparallel helices between *β*2 and *β*3 (Figure 2). The small monomeric units assemble into homo‐oligomers to form a *β*_2*n*_ *α*_*n*_ barrel, and two barrels join in a head‐to‐head orientation to form the active enzyme (Lee et al., 2007).

QueF enzymes comprise two different structural architecture types: the YkvM subfamily enzymes typical in *B. subtilis* (*bs*QueF) and *G. kaustophilus* (*gk*QueF), and YqcD subfamily enzymes isolated from *E. coli* (*ec*QueF), *V. cholerae* (*vc*QueF), and *P. carotovorum* (*pc*QueF). (Figure 1).

**FIGURE 1:**
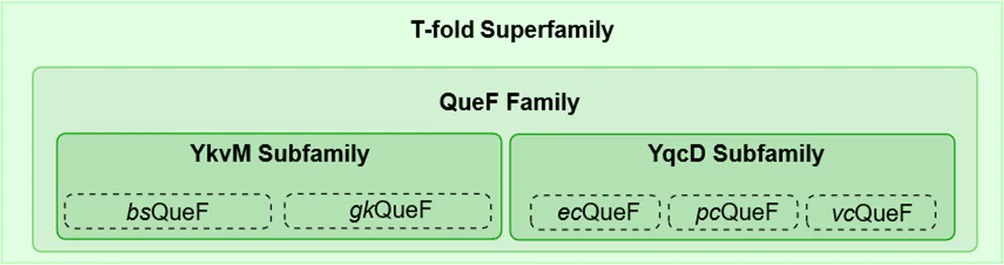
Schematic classification of QueF nitrile reductases within the T‐fold superfamily. The QueF family comprises two major subfamilies, YkvM (unimodular, e.g., *bs*QueF, *gk*QueF) and YqcD (bimodular, e.g., *ec*QueF, *pc*QueF, *vc*QueF), shown with representative enzymes.

In terms of structural composition, both subfamilies share the same QueF modules nestled within helical structures bordering the surrounding active site (Mohammad et al., 2017). Furthermore, the YkvM subfamily exhibits sequence similarity to the C‐terminal region of the T‐fold domain present in YqcD enzymes (Swairjo et al., 2005), possibly indicating an ancestral event of duplication and fusion (van Lanen et al., 2005).

Enzymes belonging to the YkvM‐type adopt a unimodular structure of about 160 residues where 10 identical domains assemble into a homodecameric state: the domains form a pentameric arrangement which further dimerises to form a decameric scaffold (dimer of pentamers) (Chikwana et al., 2012). These enzymes were initially proposed to form a homododecamer, interpreted as a dimer of hexamers (Swairjo et al., 2005). Closer crystallographic analysis, however, revealed five monomers in the asymmetric unit, and application of crystallographic two‐fold symmetry produced the full oligomer as a decamer composed of two pentameric rings arranged in a head‐to‐head formation (Figure 2b) (Chikwana et al., 2012). Each pentamer is a cyclic assembly of individual (*β*_*2*_ *α*)_2_‐barrel‐like subunits. At the single‐subunit level, the core T‐fold consists of a twisted antiparallel *β*‐sheet packed against two antiparallel *α*‐helices, followed by an additional helix and a short C‐terminal extension of ~10 residues.

**FIGURE 2:**
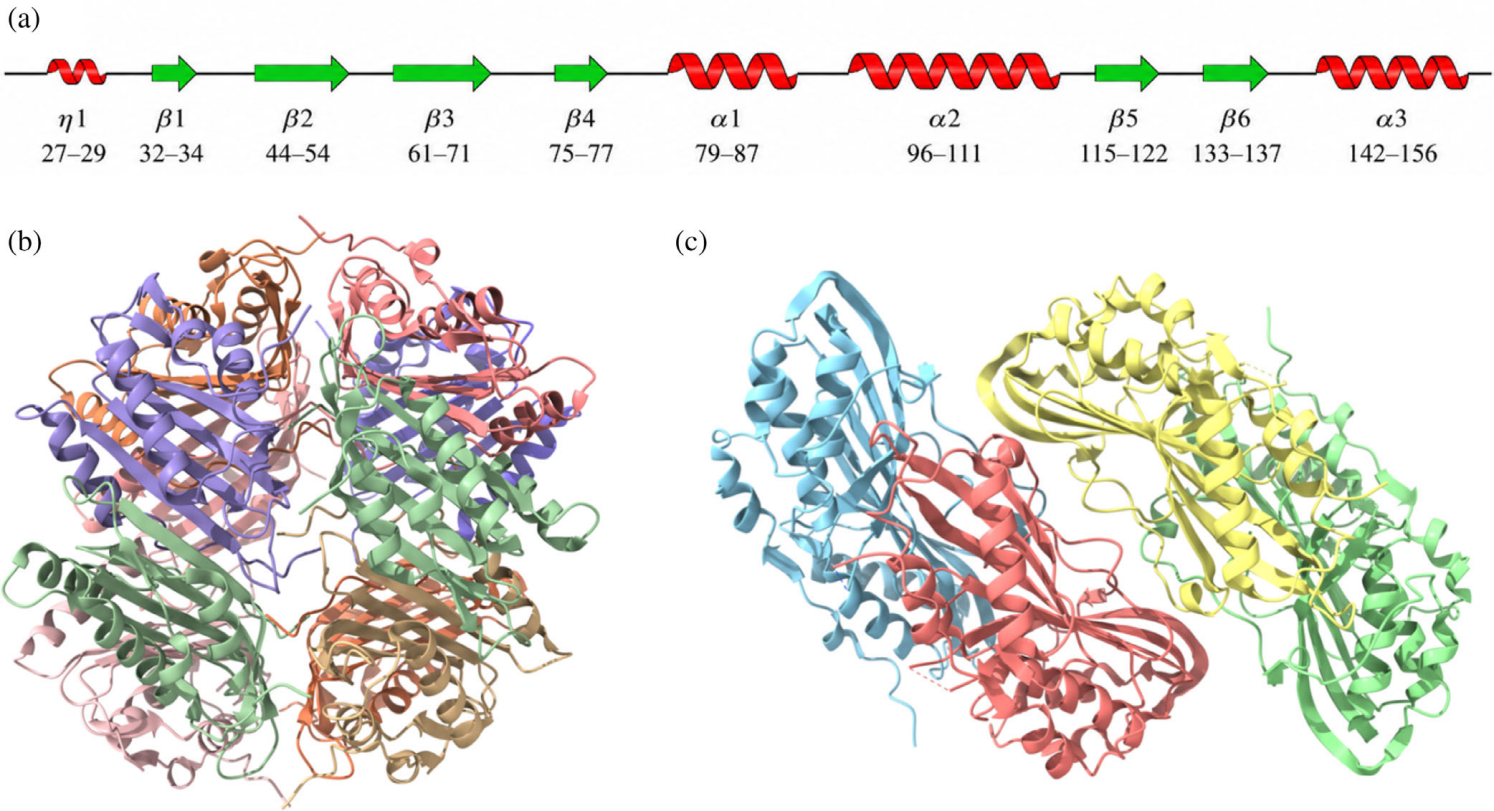
1D secondary structure map and crystal structures of QueF enzymes from the YqcD and YkvM subfamilies. (a) 1D secondary‐structure map determined for chain A of YkvM‐type *bs*QueF (PDB: 4F8B) using DSSP. Secondary‐structure elements are named according to the nomenclature used in the original study (Chikwana et al., 2012). (b) Crystal structure of the unimodular YkvM‐type *bs*QueF shown in its homodecameric state (PDB: 4F8B). (c) Crystal structure of the bimodular YqcD‐type *vc*QueF shown in its (homo)tetrameric state (PDB: 3BP1, 3RJ4). Both structures are shown in their biologically relevant assemblies.

In contrast, YqcD subfamily enzymes are established in a bimodular structure with two T‐fold modules in tandem within the same polypeptide, where 2 identical chains of about 280 residues make a homodimer with intra‐chain active sites, and thus lack the canonical quaternary T‐fold structure (Chikwana et al., 2012). Each monomer adopts a compact *α*/*β* architecture built around a seven‐stranded antiparallel *β*‐sheet, surrounded by eight *α*‐helices and connecting loops (Figure 2c) (Galperin et al., 2025; Kim et al., 2010). The N‐terminal module (residues 57–136) consists of a three‐stranded *β*‐sheet flanked by two helices and is related to the COG2904 superfamily, whereas the C‐terminal module (residues 176–271), resembling a T‐fold, consists of a four‐stranded *β*‐sheet with two helices (Kim et al., 2010). This indicates that the overall fold appears to arise from an internal duplication of a ferredoxin‐like module.

In early studies, native quaternary structures of both subfamilies were determined by size‐exclusion chromatography (SEC). The elution volume of the wild‐type and His‐6‐tagged *bs*QueF (YkvM subfamily) enzymes was measured to be 12.2 and 11.9 subunits, respectively, consistent with the molecular weight corresponding to a dodecamer structure. In contrast, the wild‐type and His‐6‐tagged *ec*QueF (YqcD subfamily) enzymes eluted with 1.8 and 1.9 subunits, respectively, indicating a homodimer structure (van Lanen et al., 2005). This observation was supported by additional SEC experiments for *vc*QueF (Kim et al., 2010), and preliminary X‐ray diffraction analysis for *bs*QueF (Swairjo et al., 2005).

## 4 THE ACTIVE SITE

QueF enzymes of both YkvM‐ and YqcD‐type subfamilies are characterized by a short highly conserved motif E(S/L)K(S/A)hK(L/Y)(Y/F/W) (where h denotes a hydrophobic amino acid) (Swairjo et al., 2005) embedded on an *α*‐helical segment, and its placement relative to other catalytic elements differs according to their modular architectures (Chikwana et al., 2012). In the unimodular YkvM QueF (*bs*QueF), the motif resides within a single T‐fold and is flanked by conserved Cys_55_ (N‐terminal side) and Glu_97_ (C‐terminal side) residues. In the bimodular YqcD QueF (*ec*QueF), the conserved Cys_190_ and Glu_230_ are spatially separated from the motif across the two modules, located on different domains rather than within a single T‐fold unit. The conserved Cys_55_ (Cys_190_) is one of the two key catalytic residues actively participating in the reduction mechanism, anchoring the substrate as an enzyme covalently bound intermediate, the other one being Asp_62_ (Asp_197_) which serves as a proton donor during the two‐hydride transfer step. PreQ_0_ is held in a productive orientation within the binding pocket by various interactions (residue numbers refer to the *bs*QueF wild‐type amino acid sequence, with the corresponding *ec*QueF residue positions shown in parentheses). Glu_97_ (Glu_230_) forms hydrogen bonds with the ring nitrogen at position 1 (N1) and with the exocyclic amino nitrogen (N2); preQ_0_ carbonyl oxygen (O6) interacts with the backbone amide nitrogen of His_96_ (His_229_) via hydrogen bond; Glu_78_ (Glu_89_) interacts with the nitrogen at position 9 (N9) of the substrate; Val_77_ (Ile_88_, this residue is based on homology with *vc*QueF) and Ser_79_ (Ser_90_) form hydrogen bonds with N2 and the nitrogen at position 3 (N3) of preQ_0_, respectively (Figure 3) (Chikwana et al., 2012; Jung & Nidetzky, 2018). Apart from hydrogen bonding interactions, hydrophobic/π‐π stacking contacts are established between preQ_0_ and Phe_33_, Phe_95_, and Ile_130_ (Phe_228_, Leu_191_, Ile_192_) to keep the substrate in place. A summary of the interacting residues anchoring preQ_0_ in place across reported NiREDs is shown in Table 1. During catalysis, both QueF subfamilies sequester the reactive thioimidate intermediate: in YkvM, preQ₀ covalent binding drives an inter‐subunit closure of the site, while in YqcD a gating loop and wall residues seal the intra‐chain pocket to exclude bulk water.

**FIGURE 3:**
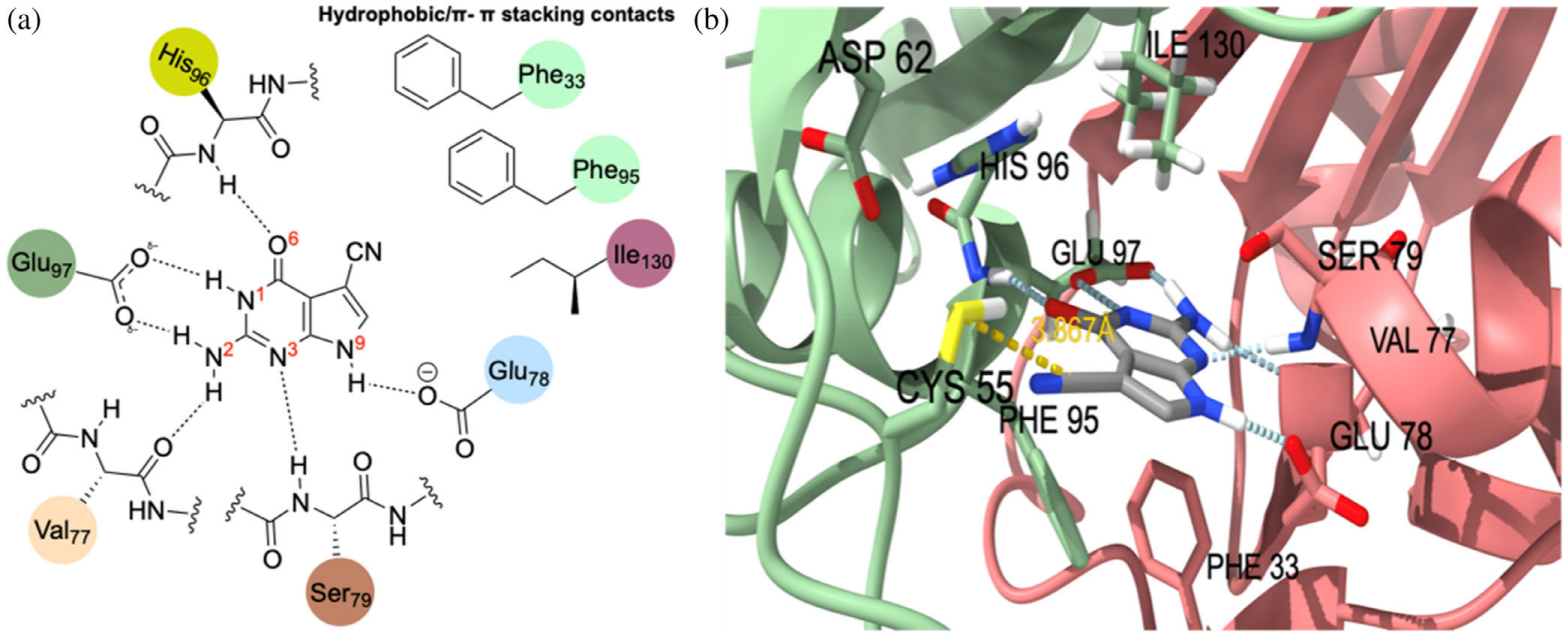
Non‐covalent interactions anchoring preQ_0_ within the *bs*QueF active‐site pocket prior to nucleophilic attack by Cys55. (a) Two‐dimensional representation of hydrogen‐bonding interactions between preQ_0_ and *bs*QueF. His96, Val77, and Ser78 engage in backbone‐mediated hydrogen bonding, while Glu97 and Glu78 interact via sidechain functional groups. (b) Three‐dimensional representation of the active site highlighting hydrogen‐bonding interactions (blue dashed lines). The distance between the preQ_0_ nitrile carbon and the sulfur atom of Cys55 is indicated (yellow dashed line) and reported in angstroms (Å).

**TABLE 1:** Overview of active site substrate‐binding residues, indicating the position of preQ_0_ contacted by each residue.

	*bs*QueF	*gk*QueF	*vc*QueF	*ec*QueF	*pc*QueF
2‐amino (N2)	Val77, Glu97		Ile93 (Val93),^a^ Glu234	Ile88, Glu230		
6‐oxo (O6)	His96		His233	His229		
Pyrimidine (N1, N3)	Glu97, Ser79		Glu234, Ser95	Glu230, Ser90, Leu191^b^		
Pyrrole (N9)	Glu78		Glu94, Ser95	Glu89, Ile192^b^		
Van der Waals/π–π stacking^c^	Phe95, Phe33, Ile130		Phe232	Phe228		

^a^ Ile93 is sometimes substituted by Val93.

^b^ Only reported for *ec*QueF.

^c^ Residues proposed to form Van der Waals or π–π stacking interactions.

Sequences of the five most studied QueF enzymes were aligned and compared to illustrate the conserved QueF motif and the key catalytic and binding residues reported for this enzyme family, revealing a high degree of conservation across subfamilies (Figure 4 and Table 2). The catalytic Cys55 in *bs*QueF and *gk*QueF, Cys190 in *ec*QueF and *pc*QueF, and Cys194 in *vc*QueF occupy an equivalent position in the active site and function as the nucleophile forming the covalent thioimidate intermediate with preQ_0_. The conserved Asp62 in *bs*QueF and *gk*QueF, Asp197 in *ec*QueF and *pc*QueF, and Asp195 in *vc*QueF, likewise play a crucial role in the catalytic pathway, stabilizing the covalent intermediate and relaying protons into the active site. Their conservation underscores the evolutionary importance of these residues in maintaining QueF activity and supports the currently accepted catalytic mechanism. Although the active sites of *gk*QueF and *pc*QueF have not yet been structurally resolved in detail, their strong sequence similarity to *bs*QueF (for *gk*QueF) and *ec*QueF (for *pc*QueF) allows to use the latter as structural and mechanistic references.

**FIGURE 4:**
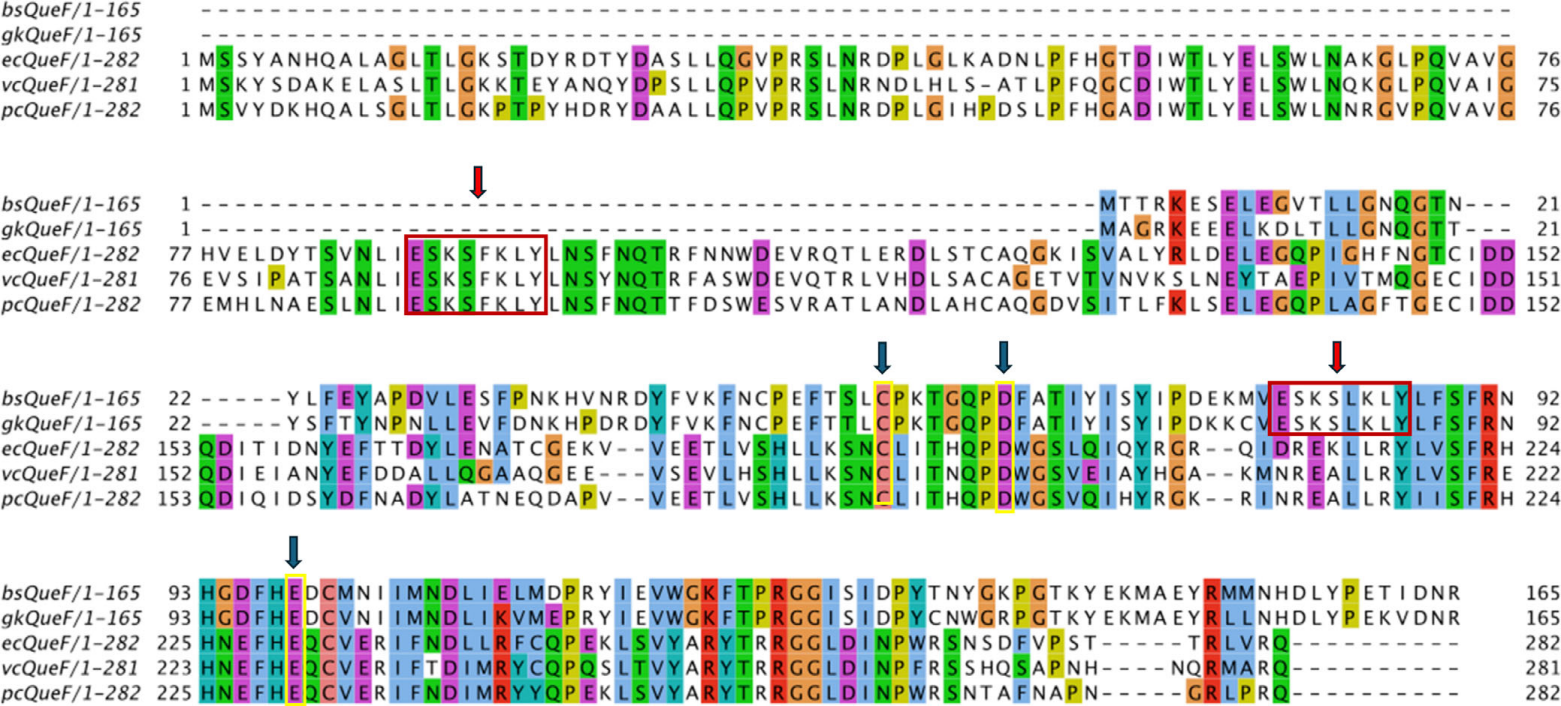
Sequence alignment of QueF enzymes from representative organisms highlighting conserved QueF motifs and key catalytic residues. Red arrows indicate the position of the conserved QueF motif in YqcD and YkvM enzymes. Blue arrows mark the invariant Cys, Asp, and Glu residue conserved across both subfamilies. Due to alignment‐based numbering, *vc*QueF Cys194 appears as Cys190 in the multiple sequence alignment.

**TABLE 2:** Summary of key catalytic and binding residues in model QueF enzymes.

	Catalytic residues	Interacting residues^e^
*bs*QueF (Chikwana et al., 2012)	Cys55, Asp62, His96^b^	Phe33, Val77, Glu78, Ser79, Phe95, Glu97, Ile130
*gk*QueF^a^ (Wilding et al., 2012)		
*ec*QueF^a^ (Jung et al., 2016; Jung & Nidetzky, 2018; Kim et al., 2010)	Cys190, Asp197, His229^b^	Trp57^d^, Ile88^c^, Glu89, Ser90, Leu191^d^, Ile192^d^, Trp198, Phe228^d^, Glu230, Arg258^d^
*pc*QueF^a^ (Li et al., 2016)		
*vc*QueF (Kim et al., 2010)	Cys194, Asp201, His233^b^	Trp62, Ile93, Glu94, Ser95, Lys96, Thr197, Phe232, Glu234, Arg262, Ile267

^a^ Crystal structures are not available; therefore, homology models were used in the cited studies.

^b^ The backbone amide of the corresponding His also participates in substrate‐binding interactions.

^c^ Residue reported to participate in binding only for *pc*QueF.

^d^ Residue reported to participate in binding only for *ec*QueF.

^e^ Listed interactions include residues that contact preQ_0_ directly and residues that contribute to the active‐site lining.

## 5 REACTION MECHANISM

As previously highlighted, the conserved cysteine and aspartic acid residues have long been recognized as the key catalytic residues responsible for the reduction of the natural substrate preQ_0_. Although both subfamilies share the core chemistry of the reduction, structural differences stand out given the modular differences between the YkvM and YqcD variants. To date, the most comprehensive mechanistic reports have focused on *bs*QueF (Chikwana et al., 2012), *ec*QueF and *vc*QueF enzymes (Jung et al., 2019; Kim et al., 2010), so the following mechanistic review is exemplified with these enzymes.

Recent work has demonstrated that QueF catalysis relies on a water‐mediated proton‐relay mechanism, in which the substrate functionally couples to a His‐H_2_O‐Asp bridge in bimodular YqcD‐type *ec*QueF enzymes (Scheme 3, Step 1) (Jung et al., 2019). This network functions as a proton supply and, with a positively charged His229 side chain, also contributes to the polarization of the substrate and potentially promotes hydride transfer from the incoming NADPH. Additionally, once the covalent thiohemiaminal intermediate breaks down into a noncovalently bound imine, Cys190 can be re‐protonated by Asp197, effectively reversing the initial proton transfer process (Scheme 3, Step 4).

**SCHEME 3:**
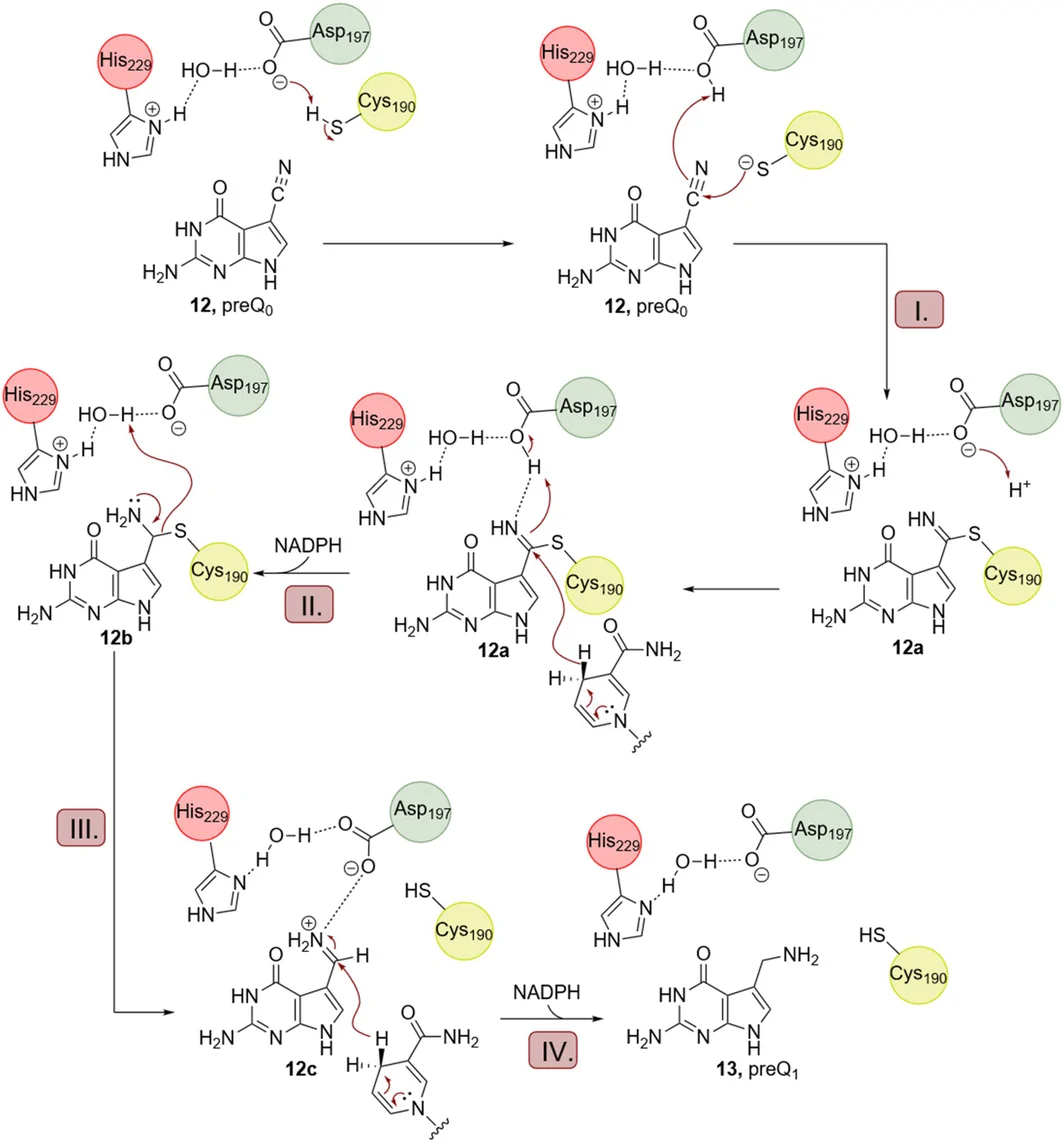
Proposed mechanism for the QueF‐catalyzed NADPH‐dependent reduction of preQ_0_ to preQ_1_ in *ec*QueF. The catalytic cycle proceeds via (I) thiolate formation at Cys190 and a covalent thioimidate intermediate, (II) hydride transfer from NADPH, (III) C–S bond cleavage, and (IV) a second NADPH hydride transfer to yield preQ_1_.

In *bs*QueF, however, structural analysis places Asp62 in direct hydrogen‐bonding contact with the covalent Cys55‐preQ_0_ thioimidate intermediate, consistent with a role as immediate proton donor, while His96 sits adjacent in the closed active site and is proposed to support this protonation step (Chikwana et al., 2012). Consequently, the presence of a His‐H_2_O‐Asp bridge has not yet been confirmed for YkvM enzymes.

The mechanism of the preQ_0_ to preQ_1_ reduction can be divided into four stages: formation of a C−S covalent bond; first hydride transfer by NADPH; cleavage of the C−S covalent bond; and second hydride transfer by NADPH (Scheme 3). Upon entering the active‐site pocket, preQ_0_ initially binds non‐covalently and is stabilized by a network of hydrogen‐bonding and hydrophobic interactions that orient the nitrile for nucleophilic attack by the catalytic cysteine (see Section 4, Figure 3b). Upon substrate binding, the active site closes, excluding bulk water while retaining the organized water relay required for proton shuttling (Chikwana et al., 2012; Jung & Nidetzky, 2018). In the first step of the reaction, the catalytic Cys190 is activated and performs a nucleophilic attack on the nitrile carbon of preQ_0_, while the conserved Asp197 donates a proton to the nitrogen (which is subsequently regenerated by the environment), generating the covalent Cys190‐substrate thioamide‐like intermediate **12a**. (Scheme 3, Step I). The intermediate is subsequently reduced by hydride transfer from NADPH, and a further protonation step involving Asp197 drives the conversion of thioimidate **12a** towards thiohemiaminal **12b**. Following these steps, cleavage of the C–S bond is facilitated by proton transfer through the His229‐H_2_O‐Asp197 network, which reprotonates the catalytic Cys190, thereby releasing the covalent adduct as a free imine intermediate **12c**. The catalytic cycle is then completed when a second NADPH delivers the final hydride, yielding preQ_1_ **13**.

## 6 THE ROLE OF NADPH

QueF catalyzes nitrile reduction via a four‐electron process in which two molecules of NADPH provide the hydride equivalents, while proton transfer is mediated by active‐site residues. The stoichiometry of NADPH consumption and the formation of a covalent thioimidate intermediate were defined by substrate titration experiments, which monitored absorbance changes at 376 nm, corresponding to thioimidate formation, and at 340 nm, reflecting NADPH oxidation (Lee et al., 2007). Beyond its role as a hydride donor, the binding mode of NADPH in YqcD‐type QueF has been the subject of extensive investigation. Early structural modeling and molecular dynamics simulations suggested that each *vc*QueF dimer contains two equivalent substrate‐binding sites, both capable of forming covalent adducts with preQ_0_ (Kim et al., 2010). However, modeling of the substrate‐bound enzyme indicated that the adenine moiety of NADPH could not be accommodated within an occupied active site. On this basis, a speculative model was proposed in which NADPH binding displaces one molecule of preQ_0_, allowing the adenine portion of the cofactor to occupy the substrate‐binding pocket while hydride transfer occurs at the opposing monomeric site (Figure 5). Subsequent experimental work employing native electrospray ionization mass spectrometry (ESI‐MS) provided direct constraints on the binding stoichiometry of both substrate and cofactor (Gjonaj et al., 2016). These studies demonstrated that two molecules of preQ_0_ bind symmetrically to the dimeric *ec*QueF enzyme, whereas only a single molecule of NADPH binds per dimer at any given time, irrespective of the order of substrate and cofactor addition. Importantly, NADPH binding was shown to be independent of substrate occupancy, ruling out a mutually exclusive substrate–cofactor binding model and contradicting the earlier proposal that NADPH binds within the preQ_0_ pocket. However, NADPH binding was also shown to accelerate thioimidate formation (~4‐fold) and to bind tightly within the enzyme‐preQ_0_‐NADPH complex during the reduction step, suggesting a regulatory binding mode that ensures complete reduction of the substrate and limits the release of partially reduced intermediates (Jung et al., 2016). Collectively, these findings support a revised mechanistic model in which NADPH binds at a different interface site, spatially separated from the two substrate‐binding pockets (Gjonaj et al., 2016). This architecture enforces functional asymmetry within the dimer, such that only one of the two bound substrate molecules is reduced at a time, consistent with half‐site reactivity. As only one NADPH molecule can bind per dimer at any given time, complete reduction of both substrate molecules requires multiple sequential NADPH‐binding events. Structural analysis suggests that the substrate‐binding pocket of *ec*QueF has a narrow entrance formed by Glu89 and neighboring residues such as Ile192 and Cys190, creating a channel through which the nicotinamide moiety of NADPH must be threaded to reach its reactive position (Jung & Nidetzky, 2018; Wilding et al., 2013). Interestingly, the C190A and D197H mutations showed no evidence for compromised NADPH binding, and both C190A and C190S variants were reported to convert preQ₀ to preQ₁ without detectable side‐product formation. Although not definitive, these findings are consistent with a reduction pathway that bypasses formation of the covalent thioimidate intermediate, for which two alternative mechanistic scenarios have been suggested (Jung et al., 2016; Jung & Nidetzky, 2018).

**FIGURE 5:**
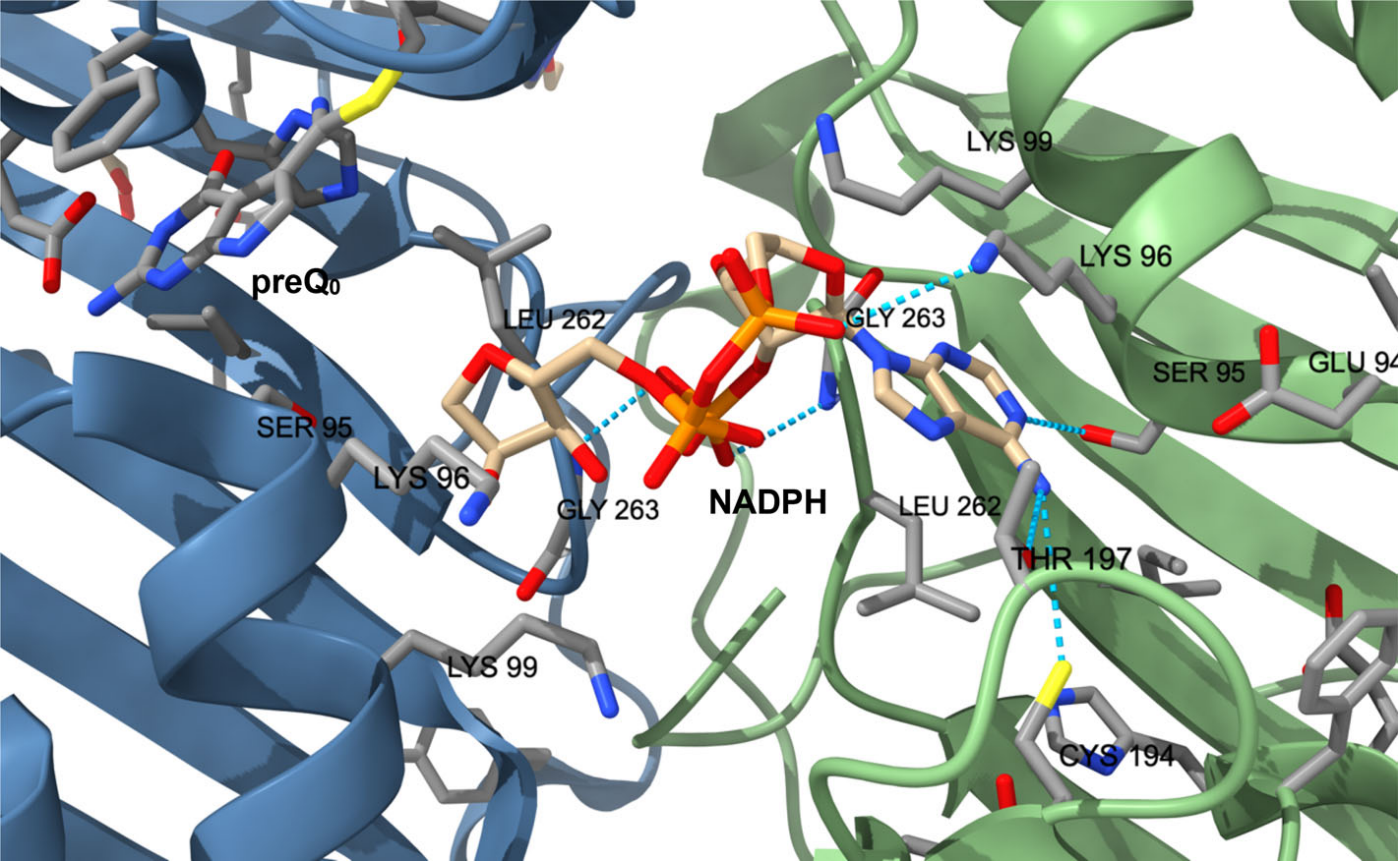
Proposed NADPH‐binding mode in *vc*QueF (PDB: 3UXJ), illustrating a model in which the adenine portion of NADPH occupies the substrate‐binding pocket. Labeled residues are those within 5 Å of the NADPH molecule in the model. Lys96 has been reported to coordinate the 2′‐phosphate group of NADPH.

## 7 MUTAGENESIS STUDIES

Both YkvM‐ and YqcD‐type QueF variants have been subjected to site‐directed mutagenesis studies, primarily to evaluate, confirm, and clarify the catalytic mechanism of the preQ₀ nitrile‐to‐amine reduction, or aiming to broaden the strict substrate specificity; where overlaps occur, those engineering‐focused results are addressed in the next section. (Section 8).

### 7.1 YkvM subfamily

In the YkvM‐type QueF enzymes, Cys55 was identified as the conserved catalytic residue. This assignment was based on the homology of *bs*QueF to *E. coli* GCYH‐I and on their similar homodecameric quaternary structures, which motivated early site‐directed mutagenesis at this position. Replacement of Cys55 with alanine or serine (C55A, C55S) resulted in the complete loss of activity towards preQ_0_ even under conditions of excess substrate and enzyme. These experiments demonstrated that the residue is indispensable for catalysis, consistent with a covalent Cys‐substrate intermediate mechanism (Lee et al., 2007; Swairjo et al., 2005). Circular dichroism (CD) and chromatographic analyses confirmed that these variants retained the native homodecameric oligomeric state, indicating that the loss of activity most likely reflected catalytic inactivation rather than structural disruption of the decamer. A second mutational series targeted the conserved intramolecular disulfide between Cys55 and Cys99 observed experimentally in *bs*QueF, which was proposed to shield the catalytic cysteine from irreversible overoxidation in vivo (Mohammad et al., 2017). Substitution of Cys99 with alanine or serine (C99A, C99S) did not impair activity, indicating that the disulfide is not part of the catalytic machinery but instead functions as a redox‐protective “off‐switch.” A similar effect was observed in *gk*QueF (Wilding et al., 2012): where the C55A variant also showed no detectable reduction of preQ₀, supporting conservation of this catalytic feature within the YkvM‐type subfamily.

Additional substitutions in *gk*QueF—H96A/F, E97A/S, and F95A—were introduced to test whether the active site could be broadened to accept preQ_0_ analogues by reducing strong polar contacts, but none of these variants showed conversion of the non‐canonical nitriles, underscoring the tight substrate specificity of YkvM‐type QueF (see Section 8.3).

### 7.2 YqcD subfamily

Systematic mechanistic studies of *ec*QueF active site have focused on the catalytic Cys190‐Asp197‐His229 triad. The catalytic nucleophile Cys190 was substituted with alanine and serine (C190A, C190S), while His229 was replaced with alanine (H229A) (Jung et al., 2019; Wilding et al., 2013). Alteration of Cys190 completely abolished activity towards preQ_0_: neither C190A nor C190S formed the characteristic thioimidate intermediate, although noncovalent substrate binding was still observed. In contrast, mutation of His229 to alanine, which has been proposed to participate in a His‐water‐Asp proton relay, did not prevent the formation of the covalent adduct (Jung et al., 2019). These results confirmed an auxiliary role for His229 in facilitating the proton transfer, but indicated that it is not strictly essential for catalysis itself, in contrast to Cys190.

Confirmation of the key role of Asp197 residue in *ec*QueF, proposed to mediate proton transfer during nitrile reduction came from target site‐directed mutagenesis studies (Jung et al., 2019; Wilding et al., 2013). Variants at this position (D197N, D197A, and D197H) resulted in complete loss of activity towards the natural substrate, consistent with the dual role of Asp197 in Cys190 activation and stabilization of early catalytic intermediates. Notably, probing Asp197 in the context of NADPH binding showed that this residue does not control cofactor association, implying an essential function in proton shuttling rather than in NADPH recognition.

A second set of mutations targeted the preQ_0_ binding pocket in *ec*QueF that physically sequester catalytic intermediates from bulk solvent: His89, Leu191, Ile192, Phe228, and surrounding positions. These residues help to orient preQ_0_ correctly and subsequently seal the active site, thereby protecting the reactive imine/thioimidate intermediate from hydrolysis. When His89 and Phe228 were substituted (E89A, E89L, F228A) (Jung & Nidetzky, 2018; Wilding et al., 2013), covalent capture of the substrate was still observed, but catalysis no longer proceeded efficiently to preQ_1_. Instead, these variants predominantly released 7‐formyl‐7‐deazaguanine, consistent with a premature solvent exposure and hydrolytic breakdown of the imine intermediate rather than completion of the full four‐electron reduction. By contrast, substitutions within the mobile structural element adjacent to the catalytic cysteine (L191A and I192A) produced mixed outcomes, with both preQ_1_ and the hydrolysis product detected (Jung & Nidetzky, 2018). This indicates that the partial opening of the active‐site lid compromises, but does not completely abolish, sequestration of the intermediate. In summary, this second set of mutations suggests that the primary role of these residues is architectural rather than catalytic.

Related insights have been obtained from the structurally homologous *vc*QueF enzyme, which has been crystallized in multiple catalytic states. Mutants such as C194S and C194A (targeting the equivalent catalytic cysteine), H233A (targeting the proposed proton‐relay histidine), and R262L were generated and structurally characterized (Jung et al., 2019; Jung & Nidetzky, 2018; Kim et al., 2010). These *vc*QueF variants were primarily used as structural models rather than as kinetically benchmarked biocatalysts: crystal structures and simulations captured the enzyme in complex with preQ_0_, covalently bound thioimidate intermediates, product (preQ_1_), and ternary complexes including NADPH. Quantum mechanics/molecular dynamics (QM/MM) analysis of C194S showed that replacement of the catalytic cysteine with serine raises the energy barrier for covalent adduct formation, rendering the pathway markedly less favorable and implying loss of activity. In agreement with this, C194A was found to bind preQ_0_ and preQ_1_ noncovalently in the crystal, but is not predicted to be catalytically competent towards the native substrate. Conversely, H233A still formed a covalent preQ_0_ adduct, consistent with an assisting (rather than strictly essential) role for His233 in proton transfer. Overall, mutagenesis of *vc*QueF supports the same mechanistic picture inferred from *ec*QueF: the catalytic cysteine and its acidic partner are essential for catalysis, whereas the nearby histidine contributes to proton movements and stabilization of intermediates.

Finally, in *pc*QueF‐type enzymes, mutagenesis was pursued not only to probe mechanism but to improve biocatalytic performance towards preQ_0_. Residues lining the active‐site pocket around the catalytic cysteine were systematically investigated with alanine scanning, excluding the catalytic triad (Cys190‐Asp197‐His229). Of the variants L37A, L95A, S99A, N101A, H224A, Q231A, D23A, N38A, N98A, F100A, Q102A, E230A, and C232A, only a subset retained measurable (albeit reduced) activity towards preQ_0_ (Zhou et al., 2018). Saturation mutagenesis at the most promising positions then yielded second‐generation mutants (N38G/E, S99G, H224V/T/N, N98G), among which H224N exhibited ~1.5‐fold higher specific activity than the wild‐type enzyme. Structural modeling suggests that, in the wild‐type enzyme, His224 forms an inter‐subunit hydrogen bond with Gln102, partially obstructing the inter‐subunit substrate channel. In the H224N variant, Asn224 instead forms an intra‐loop hydrogen‐bonding network with the backbone Phe222 and Arg223, repositioning the side chain away from the channel and thereby opening additional space for substrate and cofactor access, resulting in enhanced activity.

## 8 SUBSTRATE SELECTIVITY AND PROMISCUITY STUDIES

The highly conserved active site confers strict substrate specificity to all the characterized QueF enzymes. To date, the natural substrate preQ_0_ is the only known organic nitrile that undergoes complete conversion to the corresponding primary amine, preQ_1_. Consistent with this specificity, closely related analogues, namely the 6‐oxo substituted analogue **15**, the 2‐amino substituted analogue **19**, and the 2‐amino‐6‐oxo substituted analogue **22** have shown reduced activity (Wilding et al., 2012; Wilding et al., 2013). Nevertheless, efforts to broaden the substrate scope of these enzymes have included extensive screenings using both wild‐type and mutant QueFs. Nitrile substrates taken in consideration were grouped into two distinct classes: (i) natural substrate analogues formally correlated from preQ_0_, which show close or more distant structural resemblance; and (ii) non‐analogues aliphatic or aromatic nitriles that show essentially no resemblance to the template substrate preQ_0_ [F]. Class (i) can be further divided into 5 categories based on the original preQ_0_ functionality touched: [A] 6‐oxo substituted variants; [B] 2‐amino substituted variants; [C] 2‐amino‐6‐oxo substituted variants; [D] ring variants, in which the pyrrole and/or the pyridine portion of the preQ_0_ skeleton is altered; and [E] substrates bearing miscellaneous structural modifications. However, beside the three analogues mentioned above (compounds **15, 19, and 22**), none of the tested nitrile compounds were reported to show activity with QueF wild‐type enzymes studied.

Recent protein‐engineering efforts (Table 3, see also Table 2) on QueF have centered primarily on *gk*QueF and *ec*QueF, representing members of the YkvM‐like and YqcD‐like subfamilies, respectively, with the aim of extending their catalytic activity towards non‐natural nitriles (full list in Table 4). More recently, wild‐type *pc*QueF has been assessed against a broader panel of non‐canonical substrates. For clarity, residue numbering in this section refers to *gk*QueF, with the corresponding *ec*QueF positions indicated in parentheses (summary of the outcome in Table 5).

**TABLE 3:** Summary of mutagenesis studies on representative QueF enzymes.

	Mechanistic studies	Substrate scope studies
*bs*QueF	[C55A, C55S] (Lee et al., 2007), [C99A, C99S] (Mohammad et al., 2017)	Wild‐type *bs*QueF only (Wilding et al., 2012)
*gk*QueF (Wilding et al., 2012)	C55A	F95A, H96A, H96F, E97A, E97S
*ec*QueF	[C190A (Jung et al., 2019), E89A, E89L] (Jung & Nidetzky, 2018; Wilding et al., 2013), [H229A (Jung et al., 2019), D197N] (Wilding et al., 2013), [F228A, L191A, I192A] (Jung & Nidetzky, 2018), [C190S, D197A, D197H] (Jung et al., 2019)	[E89A, E89L, S90A, C190A (Jung et al., 2016), D197N, F228W, H229A, E230Q] (Wilding et al., 2013)
*vc*QueF (Jung et al., 2019; Jung & Nidetzky, 2018; Kim et al., 2010)	C194S, C194A, H233A, R262L	–
*pc*QueF (Li et al., 2016; Zhou et al., 2018)	L37A, L95A, S99A, N101A, H224A, Q231A, D23A, N38A, N98A, F100A, Q102A, E230A, C232A, N38G/E, S99G, H224V/T/N, N98G	Wild‐type *pc*QueF only (Li et al., 2016)

*Note*: Mutations were introduced to probe catalytic mechanism and/or expand substrate scope (see section 7 for detailed discussion).

**TABLE 4:** Overview of substrates evaluated for QueF activity in the literature.

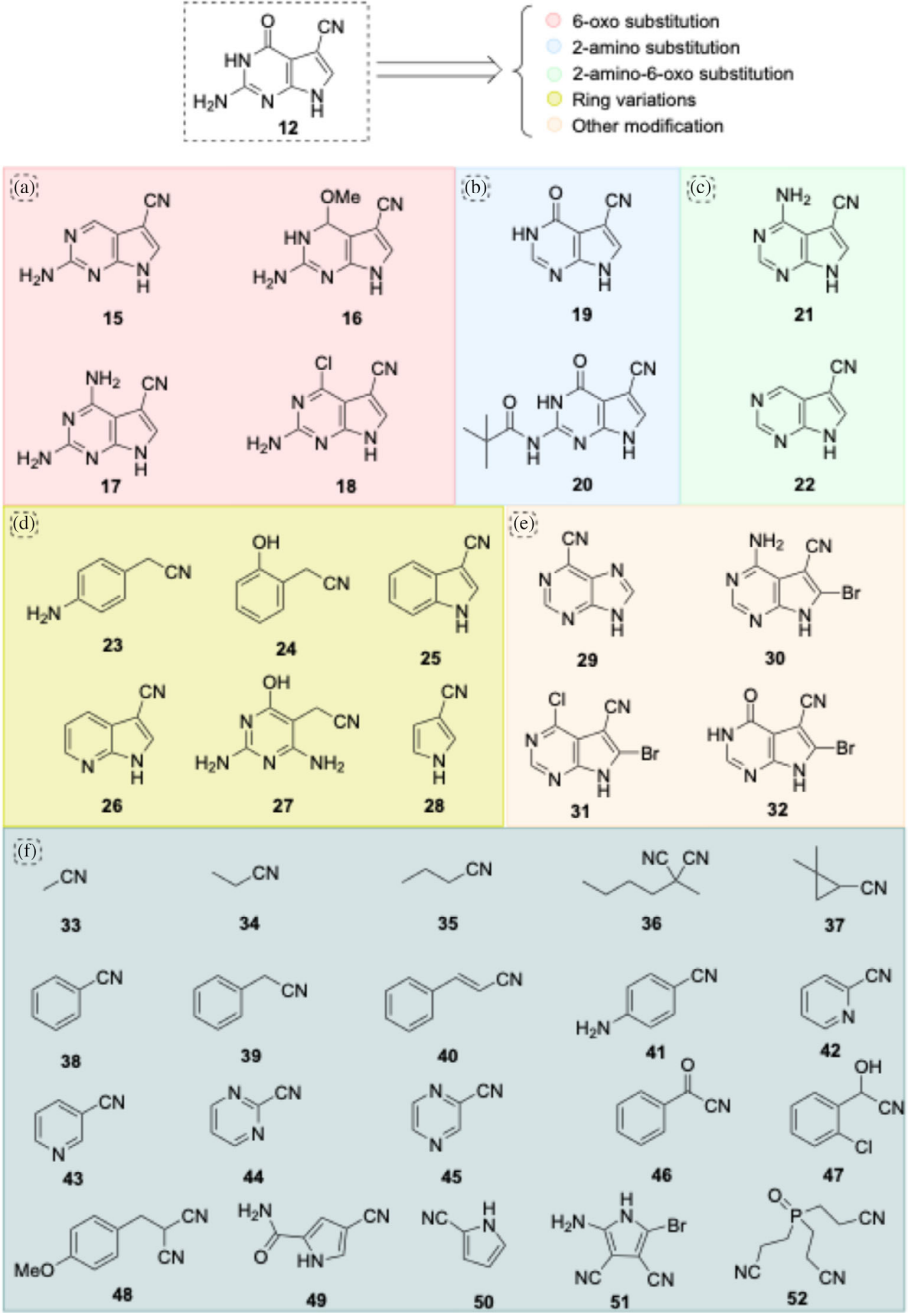

*Note*: (a)–(c) preQ_0_‐like analogues; (d) ring‐modified variants; (e) substrates bearing miscellaneous structural modifications; and (f) non‐preQ_0_‐derived substrates.

**TABLE 5:** Summary of mutagenesis studies probing the substrate scope of wild‐type and engineered *bs*QueF, *gk*QueF, *ec*QueF, and *pc*QueF.

			PreQ_0_	19	15	22	Other^c^
*bs*QueF	Wild‐type		+++	n/a	n/a	n/a	−
*gk*QueF^a^	Wild‐type		+++	++	+	n/a	−
	Glu97	Ala	++	++	+	n/a	−
		Ser	++	++	+	n/a	−
	His96	Phe	++	++	+	n/a	−
		Ala	++	++	+	−	−
	Phe95	Ala	++	++	+	−	−
*ec*QueF^b^	Wild‐type		+++	+++	−	−	−
	Glu230	Gln	++	++	−	+^a^	−
	His229	Ala	+++	+++	−	−	−
	Phe228	Trp	+++	+++	−	−	−
	Asp197	Asn	−	−	−	−	−
	Cys190	Ala	−	−	−	−	−
	Ser90	Ala	+++	+++	−	+^a^	−
	Glu89	Ala	+++	+	−	−	−
		Leu	+++	+	−	−	−
*pcQueF*	Wild‐type		+++	n/a	n/a	n/a	−

*Note*: Residue substitution and tested substrates are indicated. Activity is reported semi‐quantitatively relative to the preQ_0_ control reported in the same study: +++ = comparable to preQ_0_; ++ = lower but within the same order of magnitude; + = substantially lower; − = no detectable activity; n/a = not reported. For *ec*QueF, symbols were assigned using the conversion thresholds defined in the original study (>95%, 2–10%, <2%, 0%).

^a^ Specific activity measured by monitoring NADPH consumption at 340 nm (UV–vis) using 100 μM substrate and 250 μM NADPH in Tris–HCl buffer (pH 7.5) containing 50 mM KCl, at 55°C for 24 h. For substrate 22 with *ec*QueF mutants, the same conditions were applied at 30°C.

^b^ Activity measured with HPLC‐MS based on normalized product peak area using 45 μM enzyme, 2 mM substrate, and 0.25 mM NADPH in 100 mM Tris–HCl buffer (pH 7.5) containing 50 mM KCl and 1.15 mM TCEP, at 30°C for 24 h. An additional 4 mM NADPH was subsequently added to achieve complete conversion.

^c^ Substrate sets: *Bs*QueF, **25**, **39**, **40**, **44**, **50;** *gk*QueF, **17**, **21**, **23**–**30**, **32**, **38**–**40**, **42**–**44**, **50, 51;** *ec*QueF, **16**–**18**, **20**, **21**, **23**–**27**, **31**, **33**, **38**–**40**, **42**–**44**, **50;** *pc*QueF, **33**–**37, 41, 42, 44, 46**–**49, 52**.

### 8.1 PreQ_0_ ‐like analogues (classes A‐C)

Protein‐engineering studies have mainly focused on *gk*QueF (ykvM) and *ec*QueF (YqcD), as they represent ideal models of the two established structural lineages of QueF and crystallographically characterised in detail. The general strategy adopted was to relax the recognition elements that contact preQ_0_ at N2 and O6, His96 (His229) for O6, and Glu97 (Glu230) for N2 together with nearby supporting residues (Val77/Ser90), in the attempt to accommodate a set of analogues in a productive pose for catalysis. For instance, in *gk*QueF, the substitutions H96A, H96F, E97A, and E97S were introduced to weaken or reshape these interactions (Wilding et al., 2012). All four variants showed reduced activity on preQ_0_ relative to wild type and only residual activity on the 2‐amino analogue **19** (class B), whereas activity on the 6‐oxo analogue 1**5** (class A) was diminished even further. The corresponding *ec*QueF variants (H229A, E230Q) displayed the same trend: reduced preQ_0_ turnover and no detectable product when tested on substrates **16**, **18**, **20**, or **21** (Wilding et al., 2013). Collectively, these data point to the 6‐oxo contact (His96/His229 line) as a crucial interaction for preQ_0_‐like analogues, and they show that simple removal of individual H‐bond donors/acceptors mainly reduces activity on preQ_0_ without conferring activity on the analogues. An exception is analogue **22** (class C), which carries concurrent modifications at positions N2 and O6: *ec*QueF E230Q was still able to process this substrate, albeit at a clearly reduced rate. This illustrates that a modest change in the polar/H‐bonding environment around N2/O6 (E230Q) can restore the activity of closely analogous substrates. However, the effect remains minor, and limited to the three closest analogues (**15**, **19**, and **22**) of preQ_0_, not rendering the enzyme broadly promiscuous to non‐native substrates.

### 8.2 Ring‐modified analogues (class D)

Analogues in class [D] preserve the relative position of the N2 or N3 or N9 and 7‐cyano functionalities but alter the heteroaromatic scaffold itself. To accommodate these more flexible ring systems, mutations were directed to residues that define the walls of the active‐site pocket. In *gk*QueF these included F95A, H96A, H96F, E97A, and E97S, targeting both the polar contacts at O6‐/N2‐positions and the adjacent aromatic surface (Wilding et al., 2012); and in *ec*QueF this included E89A, E89L, S90A, F228W, H229A, and E230Q, which together modify the pocket entrance and the aromatic binding surface that engages the six‐membered face of preQ_0_ (see Sections 7.1 and 7.2) (Wilding et al., 2013). The expectation was that reducing steric bulk or removing polar side chains at these sites would create sufficient room for ring‐altered substrates while retaining the correct position of the 7‐nitrile functionality to the catalytic cysteine. Functionally, these substitutions proved to be largely non‐productive. Relaxing the ring‐binding residues consistently lowered the activity on preQ_0_ but did not yield detectable turnover on ring‐modified nitriles. This outcome is consistent with the structural picture that the QueF pocket is highly directional. The catalytic cysteine must approach the 7‐nitrile from a defined trajectory, and the aromatic surface is finely shaped around the planar deazapurine core. Once these constraints are relaxed, the enzyme loses the specific pre‐organization and the space that is created is insufficient (or incorrectly oriented) to stabilize alternative ring systems.

### 8.3 Miscellaneous nitrile substrates (class E)

Substrates in Class E retain the deazapurine core structure but either (i) reposition the nitrile functionality from C6 (substrate **12**, preQ_0_) to C7 (e.g., substrate **29**) or (ii) introduce additional substitutions at O6/N2 (carbonyl and/or amino) together with an 8‐bromo substituent (substrates **30**–**32**). For *gk*QueF, the same mutant set discussed in Sections 7.1 and 8.2 (H96A/F, E97A/S and F95A) was screened to weaken strong polar contacts within the binding pocket; however, no conversion was detected for any Class E substrate tested (Wilding et al., 2012). Similarly, *ec*QueF variants previously introduced—E89A/L, S90A, C190A, D197N, F228W, H229A, and E230Q—were designed to expand the substrate scope by mimicking ring‐stacking interactions (F228W), exchanging the amide functionality to glutamine (E230Q), and modulating side‐chain polarity/length (E89A/L and S90A), while additional mutations targeted the catalytic Cys190‐Asp197‐His229 triad (*see* Sections 7.2 and 8.2) (Wilding et al., 2013). In all cases, no conversion of substrates **29**–**32** to the corresponding primary amines was observed.

### 8.4 Non‐analogous nitrile substrates (class F)

Finally, a wide range of non‐deazapurine nitriles was assessed, primarily with wild‐type *pc*QueF and the available wild‐type and mutant *bs*QueF/*gk*QueF/*ec*QueF variants (Li et al., 2016; Moeller et al., 2013; Wilding et al., 2012; Wilding et al., 2013). Across this panel no measurable conversion was observed, confirming that QueF does not behave as a broad‐spectrum nitrile reductase but as a highly specialized preQ_0_ reductase whose catalytic machinery depends on simultaneous recognition of several heteroatoms and ring contacts. This stands in contrast to the three closest preQ_0_‐derived analogues—those lacking either the 6‐oxo group, the 2‐amino group, or both—which still showed low but detectable activity, underscoring how little deviation from the native scaffold QueF can currently tolerate. Nevertheless, the fact that these minimally perturbed analogues are processed at all suggests that targeted reshaping of the N2‐O6 sites, combined with careful preservation of the nitrile moiety, could provide a viable route to substrate broadening in future engineering attempts.

## 9 CONCLUSION AND OUTLOOK

In recent decades, nitrile reductases from the QueF family have emerged as a highly specialized biological solution to the reduction of a few organic nitriles. Mechanistic, structural, and mutagenesis studies have revealed how they achieve an unprecedented biocatalytic transformation through covalently bound intermediates mediated by a conserved cysteine–aspartate pair and a finely evolved proton relay network in a tightly water‐restricted active site environment capable of stabilizing water‐sensitive intermediates. This precisely evolved mechanism is also the principal cause of the limitations of QueF enzymes in biocatalytic applications. Because of the tight evolutionary specialization towards the sole metabolite preQ₀, QueF enzymes possess a substrate‐binding pocket specifically tuned to recognize the deazapurine scaffold through a vast conserved network of polar, aromatic, and geometric interactions. Thus, even small perturbations to substrate structure are typically poorly tolerated, and most mutagenesis efforts have compromised native activity without substantial gains in substrate scope. Future challenges will focus on overcoming this evolutionary specialization without disrupting the delicate catalytic machinery required for nitrile reduction. Conventional site‐directed mutagenesis strategies and the characterization of a limited number of naturally occurring wild‐type QueF have not been sufficient to observe any change in substrate promiscuity, indicating that more transformative approaches may be needed. These may involve directed evolution and computational or AI‐assisted approaches to reshape the overall topology and dynamics of the binding pocket. Additionally, the large‐scale exploration of the sequence space of naturally evolved QueF homologues in unexplored or extinct phylogenetic branches may result in variants with relaxed specificity. In parallel, a deeper understanding of active‐site dynamics, solvent gating, and cofactor coupling will be essential to guide these efforts. Although QueF enzymes may never give rise to generalist nitrile reductases, integrating cutting‐edge structural biology, computation, and modern enzyme engineering approaches can deliver tailored QueF‐derived catalysts for industrially relevant nitriles, helping to advance more sustainable routes to primary amines.

## AUTHOR CONTRIBUTIONS

**Chenlu Li:** Data curation; writing – original draft; visualization. **Roland Kulanda:** Writing – original draft; data curation; visualization. **Sam Staniland:** Writing – review and editing. **Daniele Castagnolo:** Supervision; writing – review and editing. **Michele Crotti:** Conceptualization; writing – review and editing; supervision; methodology; writing – original draft.

## Data Availability

Data sharing not applicable to this article as no datasets were generated or analysed during the current study.
